# Zinc tin oxide/polyvinyl alcohol nanocomposites enable sensitive pH-dependent arsenic detection by quartz crystal microbalance

**DOI:** 10.1038/s41598-025-19794-x

**Published:** 2025-09-30

**Authors:** M. M. Saadeldin, Ahmed Samir

**Affiliations:** Physics Department, Faculty of Science, Cairo University, Giza, 12613 Egypt

**Keywords:** Zinc stannate, Polyvinyl alcohol, Nanocomposites, Optical, QCM sensor, Arsenic detection, Materials science, Nanoscience and technology, Physics

## Abstract

This study presents the synthesis, comprehensive characterization, and application of zinc tin oxide/polyvinyl alcohol (ZTO/PVA) nanocomposites as sensitive platforms for arsenic detection using quartz crystal microbalance (QCM) sensors. ZTO nanoparticles were synthesized via an optimized hydrothermal method and incorporated into PVA matrices at 5, 8, and 10 wt%. Structural and optical properties were investigated using X-ray diffraction (XRD), Raman spectroscopy, scanning electron microscopy (SEM), and UV–Vis spectroscopy. XRD confirmed the formation of cubic-phase ZTO with a preferred (311) orientation, while Raman analysis verified the retention of the inverse spinel structure. SEM images revealed increased surface roughness and nanoparticle agglomeration with rising ZTO-NPs content. Optical measurements showed bandgap tunability, with the main bandgap decreasing from 5.28 eV (pure PVA) to 3.81 eV (10% ZTO/PVA), indicating strong electronic interaction between the polymer matrix and nanofiller. The presence of dual-bandgap features suggests concurrent polymer- and filler-dominated transitions. QCM sensors modified with ZTO/PVA exhibited high sensitivity toward arsenic ions in aqueous media, with optimal performance at pH 3, showing a sensitivity of 70.64 Hz/ppm and a response time of ~ 10 s. A non-linear, concentration-dependent response was observed, with enhanced sensitivity at higher arsenic concentrations, reaching 113 Hz/ppm at 10 ppm. These findings underscore the potential of ZTO/PVA nanocomposites as effective, real-time sensing materials for environmental monitoring of arsenic contamination in water systems.

## Introduction

The growing demand for advanced environmental monitoring technologies has driven the development of innovative sensing platforms capable of detecting hazardous contaminants with high precision, sensitivity, and reliability. Among these contaminants, arsenic (As), particularly in its trivalent form As (III), is of major global concern due to its widespread occurrence in groundwater and its severe health implications. Chronic exposure to arsenic is associated with serious health disorders, including arsenicosis, dermatological effects, cardiovascular complications, and various cancers^1^. As a result, leading health and environmental agencies such as the World Health Organization (WHO), United States Environmental Protection Agency (US-EPA), and the European Union (EU) have set the maximum allowable concentration of arsenic in drinking water at 10 parts per billion (ppb)^2^. This has spurred significant research into the development of reliable, rapid, and field-deployable arsenic detection technologies.

Despite advances in conventional detection techniques such as inductively coupled plasma mass spectrometry (ICP-MS) and atomic absorption spectroscopy (AAS), these methods, though highly sensitive (typically < 1 ppb), are expensive, time-intensive, and impractical for in-field deployment due to their reliance on sophisticated instrumentation and skilled personnel^3–5^. In contrast, nanomaterial-based sensors-particularly those using metal oxides have emerged as promising alternatives due to their inherent sensitivity, low cost, and potential for miniaturization^6^.

Numerous studies have explored metal oxide-based sensing systems for arsenic detection, including sensors based on ZnO, SnO_**2**_, TiO_**2**_, and various doped or composite architectures^7–10^. However, most of these systems rely on binary metal oxides and often suffer from limitations such as poor selectivity, long response times, and complicated fabrication protocols. Moreover, polymer-based nanocomposites like ZnO/PVA have shown potential^11^, but challenges remain in terms of nanoparticle dispersion, environmental stability, and limited tunability of the sensing interface under varying conditions.

To address these limitations, this study proposes a novel nanocomposite system comprising ternary zinc tin oxide (ZTO) nanoparticles (NPs) embedded within a polyvinyl alcohol (PVA) matrix as an active sensing material for quartz crystal microbalance (QCM)-based arsenic detection. ZTO is a ternary metal oxide that exhibits enhanced electronic synergy between ZnO and SnO_**2**_, resulting in improved charge transport, greater surface reactivity, and a more favorable band structure for sensing applications^12–14^. Unlike its binary counterparts, ZTO provides a tunable bandgap and better electronic conductivity, thereby enabling more efficient analytic-sensor interaction.

In this work, ZTO nanoparticles were synthesized via a controlled hydrothermal method and homogeneously dispersed within a PVA matrix to fabricate flexible and pH-responsive thin films. These films were deposited on QCM electrodes to evaluate their arsenic sensing performance under various environmental pH conditions. The structural and morphological characteristics of the ZTO/PVA nanocomposites were systematically characterized using X-ray diffraction (XRD), Raman spectroscopy, scanning electron microscopy (SEM), and UV–Vis spectroscopy. Importantly, the pH-dependent response of the sensing films was investigated in detail, considering the well-known speciation behavior of arsenic in aqueous systems.

The previous studies have reported arsenic detection using either binary metal oxide sensors or metal oxide polymer nanocomposites, this work uniquely integrates pH-tunable ZTO-NPs with PVA to fabricate flexible, environmentally responsive sensing layers for QCM-based detection of As (III)^15–17^. The novelty of this study lies in the synergistic advantages offered by the ternary ZTO system, which surpasses traditional binary oxides (ZnO or SnO_**2**_) in terms of interfacial charge transfer, electronic conductivity, and analyze interaction. By embedding these nanoparticles in a PVA matrix, the resulting nanocomposite enables enhanced dispersion, mechanical flexibility, and environmental stability. Moreover, the sensor’s performance is optimized under varying pH conditions, reflecting the real-world speciation behavior of arsenic in aqueous systems and allowing dynamic, pH-responsive detection. Coupled with the real time, label-free and rapid detection capabilities of the QCM platform, this system offers a portable, cost-effective alternative to conventional analytical techniques such as ICP-MS and AAS, particularly suitable for field applications and resource-limited settings.

Under optimal acidic conditions (pH 3), the ZTO/PVA-QCM sensor demonstrated a sensitivity of 113 Hz/ppm and a detection time of approximately 10s. The system offers significant advantages in terms of simplicity, reusability, and field applicability, especially in low-resource settings. Therefore, this work establishes a new direction for low-cost, nanocomposite-based arsenic sensors that are adaptable, scalable, and environmentally responsive.

## Experimental materials and methods

### Materials and synthesis of ZTO nanoparticles (ZTO-NPs) via hydrothermal method

High purity zinc acetate dihydrate (Zn (CH_**3**_COO)_**2**_·2H_**2**_O, 99.99%, Sigma-Aldrich) and tin(II) chloride dihydrate (SnCl_**2**_·2H_**2**_O, 99.99%, Sigma-Aldrich) were used as metal precursors for the synthesis of zinc tin oxide nanoparticles (ZTO-NPs). Separate 0.1 M aqueous solutions of each precursor were prepared in 30 mL of deionized water and stirred continuously at 500 rpm at room temperature. These solutions were then mixed in a stoichiometric ratio, and the pH was carefully adjusted to 8.5 using 1 M NaOH to promote optimal nucleation conditions.

The homogeneous precursor mixture was transferred to a 100 mL Teflon lined stainless steel autoclave, filled to 70% capacity, and subjected to hydrothermal treatment at 180 °C for 24 h. After cooling naturally to room temperature, the resulting precipitate was collected by centrifugation at 8000 rpm for 20 min.

The collected ZTO-NPs were washed five times with deionized water and three times with anhydrous ethanol to remove residual by-products. Ultra-sonication (40 kHz, 100 W, 5 min per cycle) was applied between washings to minimize agglomeration and improve purification. The purified powder was vacuum-dried at 60 °C for 48 h and subsequently annealed at 700 °C for 5 h in a muffle furnace (heating rate: 5 °C/min) to enhance crystallinity and phase purity.

### Fabrication of ZTO/PVA nanocomposite films

Polyvinyl alcohol (PVA, Mw = 89,000–98,000 g/mol, Sigma-Aldrich) was used as the polymer matrix due to its excellent film-forming ability, chemical resistance, and optical transparency. A 10 wt% PVA solution was prepared by dissolving the polymer in deionized water at 80 °C with vigorous stirring (700 rpm) for 3 h, followed by cooling to room temperature under gentle agitation.

Three nanocomposite formulations were prepared with varying ZTO loadings: S_**1**_ (5 wt %), S_**2**_ (8 wt %), and S_**3**_ (10 wt %). For each formulation, the calculated amount of ZTO-NPs was gradually added to the PVA solution under probe sonication (20 kHz, 60% amplitude, 5 s on /2 s off pulse mode) for 30 min in an ice bath to prevent thermal degradation. The dispersions were visually monitored for at least 2 h to ensure colloidal stability and absence of sedimentation.

The well-dispersed ZTO/PVA suspensions were cast onto leveled polystyrene Petri dishes (10 cm diameter) using a fixed volume of 20 mL per sample to ensure consistent film thickness. The films were dried in a humidity-controlled chamber (60 °C, 50% RH) for 48 h. Final thicknesses were measured using a digital micrometer and found to be uniform (120 ± 5 μm) across all samples. The dried films were stored in a desiccator until further use.

### Schematic of experimental workflow

**Fig. 1:**
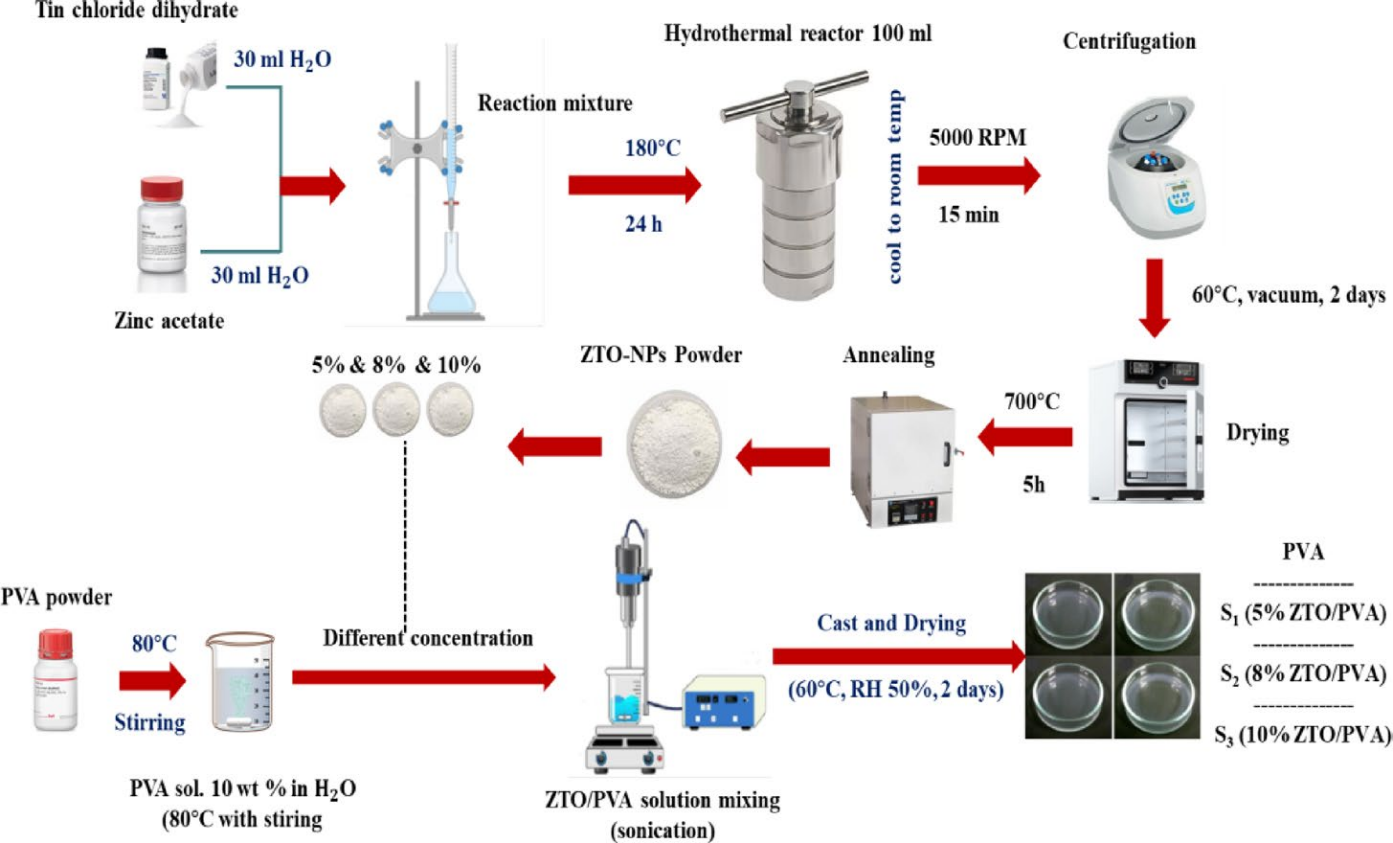
Experimental workflow for ZTO-NPs Synthesis and preparation of ZTO/PVA nanocomposites.

Figure 1 presents the schematic workflow for the synthesis of ZTO-NPs via the hydrothermal method and the subsequent fabrication of ZTO/PVA nanocomposite films with controlled nanoparticle loading.

### Characterization techniques

A comprehensive suite of analytical techniques was employed to characterize the structural, morphological, optical, and surface properties of the synthesized ZTO nanoparticles (ZTO-NPs) and their PVA-based nanocomposites.

#### X-ray diffraction (XRD)

The crystalline structure of ZTO-NPs and ZTO/PVA composite films were investigated using a Rigaku Ultima III X-ray diffractometer equipped with Cu Kα radiation (λ = 1.5406 Å). Diffraction patterns were recorded over a 2θ range of 20–80° with a step size of 0.02° and a scan rate of 2 min^−1^. Phase identification was performed by comparing the diffraction peaks to standard reference patterns, while the average crystallite size was estimated using the Scherrer equation.

#### Raman spectroscopy

Raman spectra were acquired using a DXR3 Raman Microscope (Thermo Fisher Scientific, USA) to evaluate vibrational modes and confirm structural characteristics. A 532 nm excitation laser with power limited to < 5 mW was used to minimize sample heating. Spectra were collected in the range of 100–1000 cm⁻^1^ at room temperature (23 ± 2 °C), with a spatial resolution of 1 μm and a confocal depth of 2 μm. Each spectrum was obtained with an acquisition time of 60 s and averaged over three accumulations to enhance signal-to-noise ratio. All measurements were performed under ambient controlled conditions in compliance with 21 CFR Part 11.

#### Scanning electron microscopy (SEM)

Surface morphology and microstructural features were observed using a FEI Inspect S50 scanning electron microscope. Prior to imaging, all samples were sputter-coated with a ~ 10 nm gold layer to improve electrical conductivity and suppress charging artifacts. Images were captured at magnifications ranging from 1000 × to 50,000 × using an accelerating voltage of 15 kV and a working distance of ~ 10 mm. Both secondary electron (SE) and backscattered electron (BSE) detectors were employed to obtain detailed morphological contrast.

#### UV–Vis–NIR spectroscopy

The optical characteristics of ZTO and ZTO/PVA films were examined using a Jasco V-770 UV–Vis–NIR spectrophotometer over the spectral range of 190–1000 nm with a 1 nm resolution. Diffuse reflectance spectra were collected using an integrating sphere, with barium sulphate serving as the reflectance standard. The optical bandgap was estimated using Tauc plot analysis, assuming direct allowed transitions.

#### Quartz crystal microbalance with dissipation monitoring (QCM-D)

Arsenic sensing performance was assessed using a QCM-D system (Q Sense, Biolin Scientific, USA). Gold-coated AT-cut quartz crystals (5 MHz fundamental frequency) were cleaned using piranha solution (3:1 H_**2**_SO_**4**_:H_**2**_O_**2**_; CAUTION: highly corrosive), rinsed thoroughly with ultrapure deionized water (18.2 MΩ·cm), and dried under nitrogen gas. The cleaned surfaces were functionalized via drop-casting of ZTO-NPs. Measurements were conducted at 25 °C with a constant flow rate of 100 μL/min. Changes in resonance frequency (Δf) and energy dissipation (ΔD) were monitored simultaneously at multiple overtones (n = 3, 5, 7, 9, 11, 13) to analyze mass adsorption behavior and viscoelastic properties of the sensing layer during arsenic exposure.

## Result and discussion

### X-ray diffraction (XRD)

**Fig. 2:**
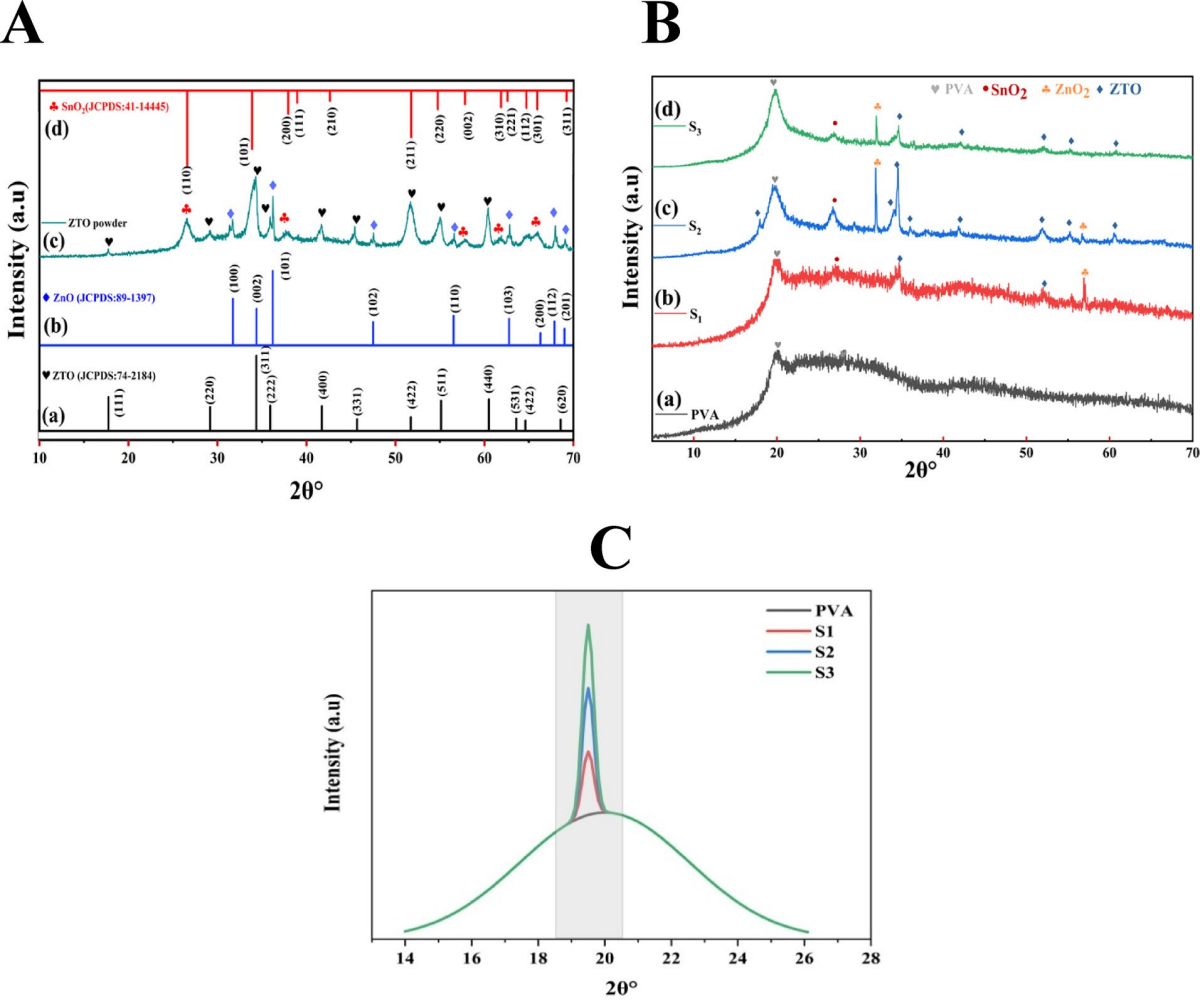
(**A**) XRD patterns shows (**a**) standard JCPDS peaks for ZTO, (**b**) ZnO, (**c**) synthesized ZTO-NPs, and (**d**) SnO_**2**_. (**B**) XRD shows (**a**) pure PVA, (**b**) S_**1**_ (5% ZTO/PVA), (**c**) S_**2**_ (8% ZTO/PVA), and (**d**) S_**3**_ (10% ZTO/PVA) nanocomposites. (**C**) XRD shows how the sharp peaks added on top of the broad PVA amorphous halo around ~ 19.5° for S_**1**_, S_**2**_, and S_**3.**_

X-ray diffraction analysis was performed to characterize the crystallographic structure and phase composition of the synthesized ZTO-NPs and ZTO/PVA nanocomposite films. Figure 2A present the XRD patterns of ZTO synthesized via hydrothermal method compared with pure ZnO and SnO_**2**_ pure. The ZTO-NPs exhibited distinct diffraction peaks at 17.72°, 29.12°, 34.29°, 35.91°, 41.66°, 45.40°, 51.67°, 55.02°, and 60.39°, corresponding to the (111), (220), (311), (222), (400), (331), (422), (511), and (440) crystal planes, respectively. These peaks align precisely with the standard JCPDS card (74–2184), confirming the formation of cubic spinel phase of Zn_**2**_SnO_**4**_ (space group *Fd*3̅*m*, No. 227), with a preferential orientation in the (311) direction^14,18^.

The XRD pattern of pure ZnO displayed peaks at 31.68°, 36.23°, 47.53°, 56.57°, 62.85°, 67.94°, and 69.06°, indexed to the (100), (101), (102), (110), (103), (112), and (201) planes of the hexagonal wurtzite structure (space group *P6₃mc*, No. 186) as per JCPDS card (89–1397), with a preferred orientation along the (101) plane. Meanwhile, SnO_**2**_ displays prominent reflections at 26.52°, 37.90°, 57.81°, 61.78°, and 65.74°, indexed to the (110), (200), (002), (310), and (301) planes of the tetragonal rutile phase (JCPDS card 88–0287, space group *P4₂/mnm*, No. 136), showing preferential growth along the (110) direction^19^.

The absence of extraneous diffraction peaks in the ZTO-NPs pattern confirmed the high purity of the synthesized material and the successful integration of ZnO and SnO_**2**_ into the cubic ZTO structure. These results validate the synthesis strategy and align with prior reports on phase-pure ZTO nanomaterials^20^.

Figure 2B illustrates the XRD patterns of pure PVA and ZTO/PVA nanocomposite films with increasing ZTO loading (samples S_**1**_, S_**2**_, and S_**3**_). Pure PVA shows a broad, low-intensity diffraction halo centered around 19.5°, characteristic of its semi-crystalline nature^21^. Upon incorporation of ZTO-NPs, the diffraction peaks become sharper and more intense, particularly at higher nanoparticle content. These peaks are superimposed on the amorphous PVA background and correspond to the characteristic ZTO crystalline planes, confirming successful nanofiller dispersion and strong structural interaction between the components.

A noticeable shift of diffraction peaks toward lower 2θ values is observed with increasing ZTO content, suggesting lattice expansion, likely caused by interfacial strain between the nanoparticles and the polymer matrix^22,23^. In addition, the progressive increase in peak intensity and sharpness reflects a substantial enhancement in the overall crystallinity of the nanocomposites.

The crystallinity enhancement factor (CEF) was calculated by comparing the intensity of the principal crystalline peak of each ZTO/PVA nanocomposite sample (S_**1**_, S_**2**_, S_**3**_) to that of pure PVA as shows in Fig. 2C, according to the following relation:

1 $$CEF = \frac{{I_{composite} }}{{I_{PVA} }}$$

where I _composite_ is the intensity of the most prominent crystalline peak in the nanocomposite and I_PVA_ is the intensity of the broad halo peak of pure PVA around 19.5°. The calculated crystallinity enhancement factors, relative to pure PVA, were 1.38 for S_**1**_, 4.02 for S_**2**_, and 4.79 for S_**3**_. This improvement is attributed to the nucleating effect of ZTO-NPs, which induce more ordered alignment and packing of PVA chains in proximity to the nanoparticle surface^24,25^.

The observed trend clearly indicates that increasing ZTO content not only promotes crystal growth but also enhances the degree of structural organization within the polymer matrix. This behavior is most likely driven by strong interfacial interactions, including hydrogen bonding and chain entanglement, which limit the mobility of PVA chains and favor the formation of more regular and tightly packed domains^26,27^. Such synergistic interactions between ZTO-NPs and the PVA matrix are crucial for improving the nanocomposites’ physical integrity and functional performance in sensing applications^28,29^.

Although PVA is semi-crystalline and typically presents broad amorphous halos, the incorporation of ZTO nanoparticles and the improved processing gradient allowed the crystalline features of ZTO to dominate in the updated patterns (Fig. 2B). Structural parameters for the nanocomposites were extracted using the well-defined crystalline peaks of the ZTO phase embedded in the PVA matrix. The following equations were used:

The crystallite sizes (D) were quantitatively estimated using the Debye–Scherrer equation from the most intense diffraction peaks corresponding to the (311) plane of ZTO^19,30^.

2 $$D_{s} = \frac{k\lambda }{{\beta \cos \theta }}$$

where k is the shape factor (0.9), λ is the X-ray wavelength (1.5406 Å), β is the full-width at half maximum (FWHM) in radians, and θ is the Bragg angle. Additional structural parameters, including microstrain (ε), lattice parameter (a), unit cell volume (V_**n**_), and dislocation density (δ), were calculated using the following equations^20,31^:

3 $$\varepsilon = \frac{\beta }{4\tan \theta }$$

4 $${\text{a}}_{{{\text{hkl}}}} {\text{ = d}}_{{\text{s}}} \sqrt {{\text{h}}^{{2}} {\text{ + k}}^{{2}} {\text{ + l}}^{{2}} }$$

5 $${\text{V}}_{{\text{u}}} { = }\;{\text{a}}_{{{\text{hkl}}}}^{{3}}$$

6 $${\updelta }\;{ = }\frac{{1}}{{{\text{D}}_{{\text{s}}}^{{2}} }}$$

Table 1 summarizes the calculated structural parameters for the ZTO/PVA nanocomposite films. Notable increases in crystallite size from 28.57 nm (pure ZTO-NPs) to 37.45 nm (S_**2**_) alongside a concurrent reduction in microstrain (from 0.43 × 10^–2^ to 0.33 × 10^–2^) suggest relaxation of internal stresses upon nanocomposite formation. The minor reduction in lattice parameters (**a**_hkl_) and unit cell volume (**V**_u_) implies partial lattice distortion at the nanoparticle-polymer interface effects. Furthermore, the significant decrease in dislocation density (from 12.26 × 10^−4^ to ~ 7.56 × 10^–4^ nm^−2^) indicates that increasing ZTO-NPs loading enhances crystal quality, minimizes lattice imperfections, and thereby improves structural integrity, which can translate into superior functional performance.

**Table 1:** Illustrated structural parameters derived from XRD analysis for ZTO/PVA nanocomposite films.

Samples	D(nm))	${\upvarepsilon }$($\times$ 10^−2^)	a_hkl_ (Å)	V_u_(Å)^3^	${{\varvec{\updelta}}}$(nm)^−2^ $\times$ 10^–4^
ZTO-NPs	28.57	0.43	8.66	649.26	12.26
S_1_	34.77	0.35	8.56	627.22	8.27
S_2_	37.45	0.33	8.61	637.31	7.13
S_3_	36.36	0.33	8.59	633.56	7.56

Including crystallite size (D), microstrain (**ε**), lattice parameters (aₕₖₗ), unit cell volume (**V**_u_), and dislocation density (**δ**) for ZTO/PVA nanocomposite films (S_**1**_, S_**2**_, and S_**3**_).

These values, listed in Table 1, reflect how ZTO-NPs loading affect the nanocomposite’s crystallite size, internal strain, and lattice integrity. The successful shift from a predominantly amorphous to a semi-crystalline structure validates the effectiveness of gradient optimization and ZTO integration. These quantitative metrics confirm that increased ZTO-NPs content promotes nucleation and structural ordering within the PVA matrix, in agreement with reports on oxide polymer nanocomposites^24,25^.

### Raman spectroscopy analysis

**Fig. 3:**
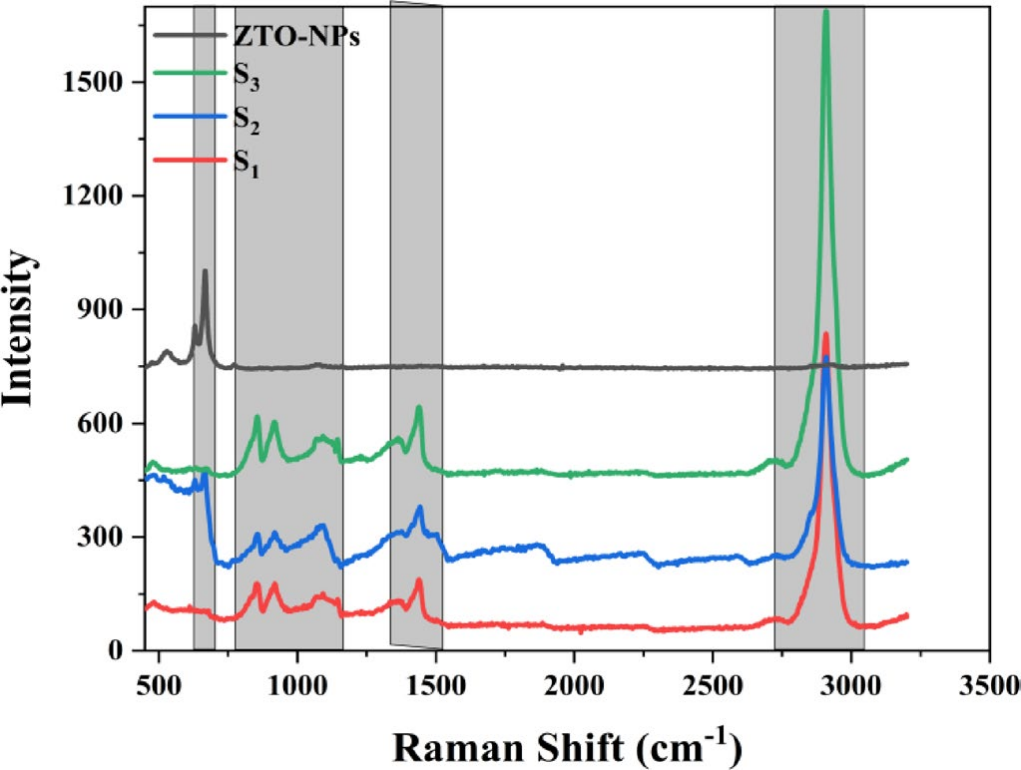
Raman spectra of pure ZTO-NPs and ZTO/PVA nanocomposite films (S_**1**_, S_**2**_, and S_**3**_).

Raman spectroscopy was performed to investigate the phase composition, crystallinity, molecular interactions, and structural quality of ZTO-NPs and their PVA-based nanocomposites. The Raman spectra of pure ZTO-NPs and nanocomposite films with increasing ZTO concentrations (S_**1**_, S_**2**_, and S_**3**_) are presented in Fig. 3.

#### Phase composition and crystallinity

The Raman spectrum of the pure ZTO-NPs exhibits multiple well-resolved peaks in the 400–800 cm^−1^ region, characteristic of the cubic inverse spinel structure of ZTO. A strong band at ~ 540 cm^−1^ corresponds to oxygen vibrations in tetrahedral coordination, while the sharp peak near ~ 675 cm^−1^ is attributed to metal oxygen (M–O) stretching vibrations in MO_**6**_ octahedral. These peaks are assigned to the F_**2**_g (2), F_**2**_g (3), and A_**1**_g modes of the spinel lattice, consistent with prior reports on phase-pure ZTO materials^32–34^. The spinel specific peaks near 540 cm^−1^ and 675 cm^−1^ match closely with those reported for hydrothermally synthesized ZTO^32,33^. Notably, no features associated with hydroxide-related impurity phases, such as ZnSn (OH)_**6**_ typically observed around 300, 374, 438, and 610 cm^−1^ were detected, confirming the high crystallinity and phase purity of the ZTO structure^35^.

#### Polymer-filler interactions and structural evolution

Upon integration of ZTO-NPs into the PVA matrix, several significant spectral modifications were observed: First retention and broadening of ZTO modes (500–800 cm^−1^) the key spinel peaks are retained in all nanocomposite samples, indicating structural preservation of the ZTO phase. However, a degree of peak broadening and reduced intensity in S_**1**_ and S_**2**_ suggests partial lattice disorder, likely due to interfacial strain and strong polymer nanoparticle interactions. The polymer-associated vibrations agree with reported PVA-based oxide nanocomposites such as ZnO/PVA and TiO_2_/PVA^36,37^. The slight red-shift and broadening of ZTO modes in S_1_ to S_3_, relative to pure ZTO, align with literature observations attributing such effects to interfacial polymer filler interactions^36,38^. Second new vibrational bands appear in the 800–1500 cm^−1^ region for all composite samples. The peaks at approximately 850, 920, 1060, and 1150 cm^−1^ can be attributed to C–C stretching, C–O–C stretching and C-O stretching vibrations of the PVA backbone. A band at ~ 1350 cm^−1^ is associated with CH₂ and O–H bending, indicative of the complex vibrational landscape of the polymer matrix. These features reflect the integration of ZTO within the PVA structure and the emergence of new interfacial modes. Third in the high-frequency region (2800–3000 cm^−1^**)** a strong broad band centered at ~ 2940 cm^−1^ was observed in all composite samples, corresponding to symmetric and asymmetric stretching of CH and CH_**2**_ groups in PVA. The increasing intensity of this band from S_1_ to S_3_ suggests enhanced polymer chain alignment and interfacial interaction as ZTO content increases^39,40^.

These spectral changes collectively reveal that ZTO loading has a pronounced effect on the molecular organization and vibrational environment within the PVA matrix, suggesting strong interfacial bonding and potential hydrogen-bonding interactions between hydroxyl groups of PVA and the ZTO surface.

#### Structural quality and interface assessment

The shaded gray regions in the Raman spectra highlight key vibrational bands that provide insights into phase quality and interface phenomena. The 500–700 cm^−1^ region captures the spinel-specific vibrations of ZTO, while the broad band at 2800–3000 cm^−1^ serves as a sensitive indicator of polymer conformation and crystallinity. The presence of sharp and well-defined peaks in both pure ZTO and the high-loading composite sample (S_**3**_) suggests improved crystallinity at higher nanoparticle concentrations. Furthermore, the flat spectral baseline of the ZTO-NPs spectrum indicates minimal amorphous content, reinforcing the high quality of the crystalline phase^41^.

In prior works on similar nanocomposites have also reported Raman peak broadening and redshifts due to strong polymer–filler interactions and interfacial strain, which supports the trends observed in this study^36,38^. Additionally, the intensity enhancement of high-frequency modes with increasing filler content is consistent with reports of nanoparticle-induced ordering in polymer chains^42,43^.

These Raman results are in good agreement with our SEM observations, which revealed increased nanoparticle clustering and surface roughness at higher ZTO loadings, suggesting stronger inter-particle interactions and reduced polymer chain mobility. Furthermore, UV–Vis absorption spectra showed a consistent red-shift and narrowing of the optical bandgap with increasing ZTO content, indicating enhanced electronic interactions between ZTO nanoparticles and the PVA matrix. The observed reduction in bandgap at higher ZTO loadings is likely associated with the increased crystallinity and aggregation detected in both SEM and Raman analyses, reinforcing the conclusion that higher ZTO content significantly alters the structural and optical properties of the nanocomposites.

**Fig. 4:**
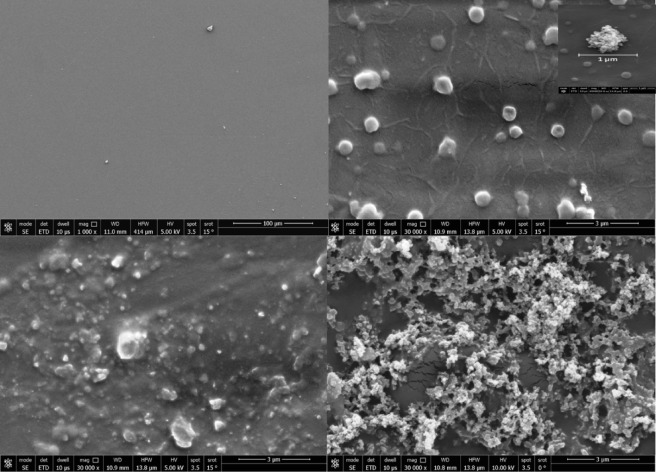
SEM micrographs of: (**a**) pure PVA, (**b**) S_**1**_ (low ZTO content), and inset dimension of the aggregates. (**c**) S_**2**_ (moderate ZTO content), and (**d**) S_**3**_ (high ZTO content). Increasing ZTO concentration leads to higher surface roughness and more pronounced nanoparticle agglomeration.

*Surface morphology *via* scanning electron microscopy (SEM)* Scanning Electron Microscopy (SEM) was utilized to evaluate the effect of ZTO-NPs incorporation on the surface morphology of polyvinyl alcohol (PVA) matrices. Figure 4 displays representative SEM micrographs of pure PVA and ZTO/PVA nanocomposites with varying nanoparticle concentrations (denoted as S_**1**_, S_**2**_, and S_**3**_). As shown in Fig. 4a, the surface of pristine PVA is relatively smooth and lacks prominent topographical features, indicative of its homogeneous polymeric structure. Upon the introduction of ZTO-NPs in sample S_**1**_ (Fig. 4b), the surface displays increased roughness with visible nanoparticle dispersion throughout the matrix. The distribution is largely uniform; however, localized nanoparticle aggregation is apparent. Preliminary particle size estimation using Image J software indicates that the aggregates in S_**1**_ range from approximately 50 to 75 nm, in agreement with previous reports on ZTO-polymer composites^44^.

With increasing the nanoparticle concentration in sample S_**2**_ (Fig. 4c), more pronounced aggregation occurs, leading to the formation of larger clusters. The average aggregate size increases to approximately 100–150 nm, indicating a decline in dispersion homogeneity as the nanoparticle loading increases^37^. In the case of sample S_**3**_ (Fig. 4d), corresponding to the highest ZTO-NPs concentration, the surface morphology becomes significantly more irregular, with dense and large agglomerates exceeding 200 nm in diameter. These findings suggest that at elevated loadings, ZTO-NPs tend to self-associate, reducing their interaction with the polymeric matrix and compromising uniform distribution^37,45^.

The progressive agglomeration observed with increasing ZTO-NPs content can be attributed to several contributing factors such as high surface energy of ZTO-NPs, promoting coalescence to reduce interfacial energy; exceeding the percolation threshold, leading to saturation of dispersion capacity within the polymer matrix, and reduced polymer-nanoparticle interaction at higher loadings due to limited chain entanglement and steric hindrance^46^.

These morphological transformations are critical, as surface topology plays a vital role in dictating the composite’s bulk properties. Increased surface roughness and nanoparticle aggregation can influence light scattering, charge transport, and mechanical reinforcement, thereby affecting the optical, dielectric, and sensing performance of the nanocomposites.

The SEM analysis demonstrates a direct correlation between nanoparticle concentration and morphological evolution in ZTO/PVA nanocomposites. As ZTO loading increases from S_**1**_ to S_**3**_, surface roughness and particle aggregation intensify, which could potentially modulate the composite’s functionality. These findings complement Raman and UV–Vis analyses, providing a comprehensive understanding of how nanoscale architecture affects macro-level properties.

### Optical properties of ZTO/PVA nanocomposites

#### UV–visible spectroscopic analysis

**Fig. 5:**
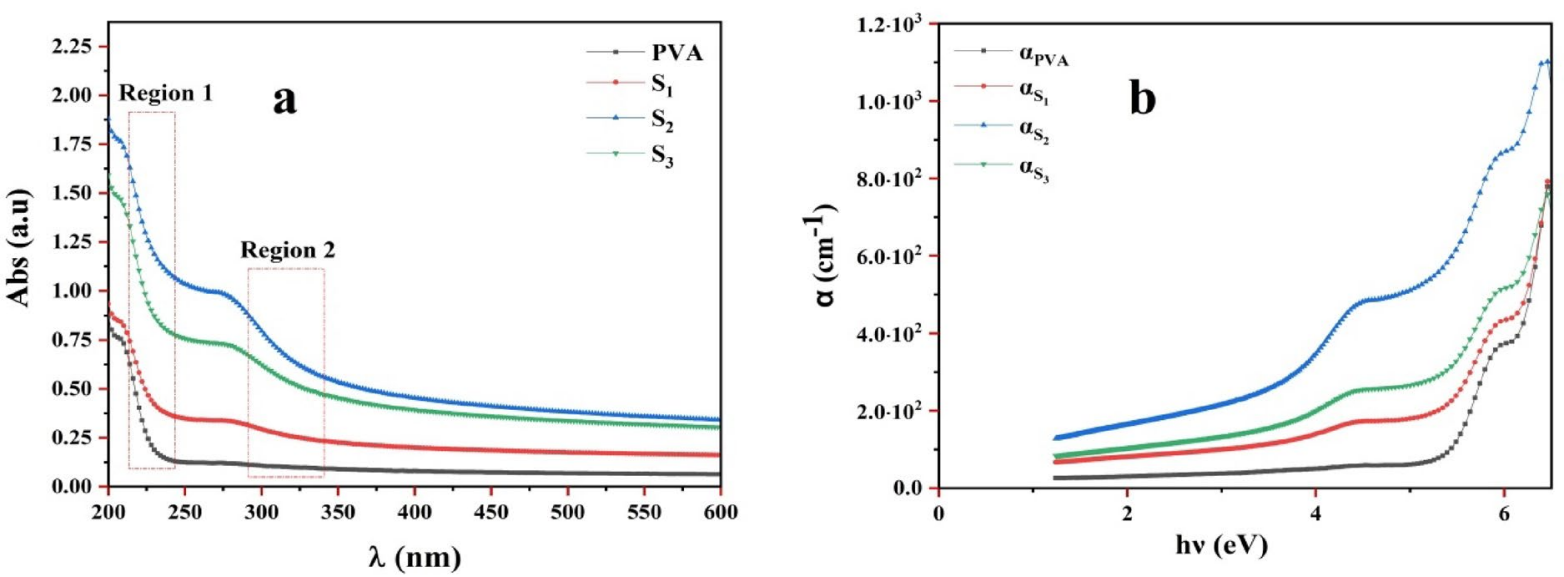
(**a**) UV–Vis absorption spectra of pure PVA, S_**1**_, S_**2**_, and S_**3**_. (**b**) Absorption coefficient (α) versus photon energy (hν), demonstrating enhanced light absorption with increasing ZTO content.

The optical behavior of the synthesized ZTO/PVA nanocomposites was investigated using UV–Vis spectroscopy to assess their electronic structure and bandgap characteristics. Figure 5a displays the absorption spectra of pure PVA and nanocomposite samples S_**1**_, S_**2**_, and S_**3**_. As expected, pure PVA exhibits negligible absorption in the UV region due to its wide bandgap. However, with increasing ZTO-NPs loading, a marked enhancement in UV absorption is observed, indicating improved optical activity and partial crystallinity in the composites^47^. This trend is consistent with the XRD results reported earlier, which confirm increased crystallinity upon nanoparticle incorporation^47,48^.

A noticeable red shift in the absorption edge with increasing ZTO content reflects the progressive modulation of the material’s band structure, attributed to enhanced charge transfer and electronic interaction between the filler and the polymer matrix^49^. The visual transformation of the films from transparent PVA to semi-transparent (S_**1**_, S_**2**_, and S_**3**_) further supports the increased light absorption. Notably, sample S_**2**_ displays higher absorbance than S_**3**_, likely due to more efficient nanoparticle dispersion, as confirmed by the SEM analysis (Sect. “Optical properties of ZTO/PVA nanocomposites”). In S_**3**_, excessive nanoparticle agglomeration impairs optical homogeneity, leading to reduced light interaction efficiency.

The absorption coefficient (α) was calculated using the Beer–Lambert law as a function of photon energy (hν) using Eq. 7.

7 $$\alpha = \frac{{{2}{\text{.302}}}}{{\text{d}}}{\text{ log}}\frac{{{\text{I}}_{{0}} }}{{\text{I}}}$$

Where d represents the sample thickness, and I_**0**_ and I denote the intensities of the incident and transmitted light, respectively^50^.

Figure 5b shows α as a function of photon energy (hν), revealing increasing optical density with ZTO content. The optical bandgap (E_**g**_) was determined using Tauc’s relation (Eq. 8).

8 $$\alpha \, h\nu = \beta \left( { h\nu - E_{g} } \right)^{\gamma }$$

For direct allowed transitions (γ = 1/2), where β is a material- specific constant^51^.

**Fig. 6:**
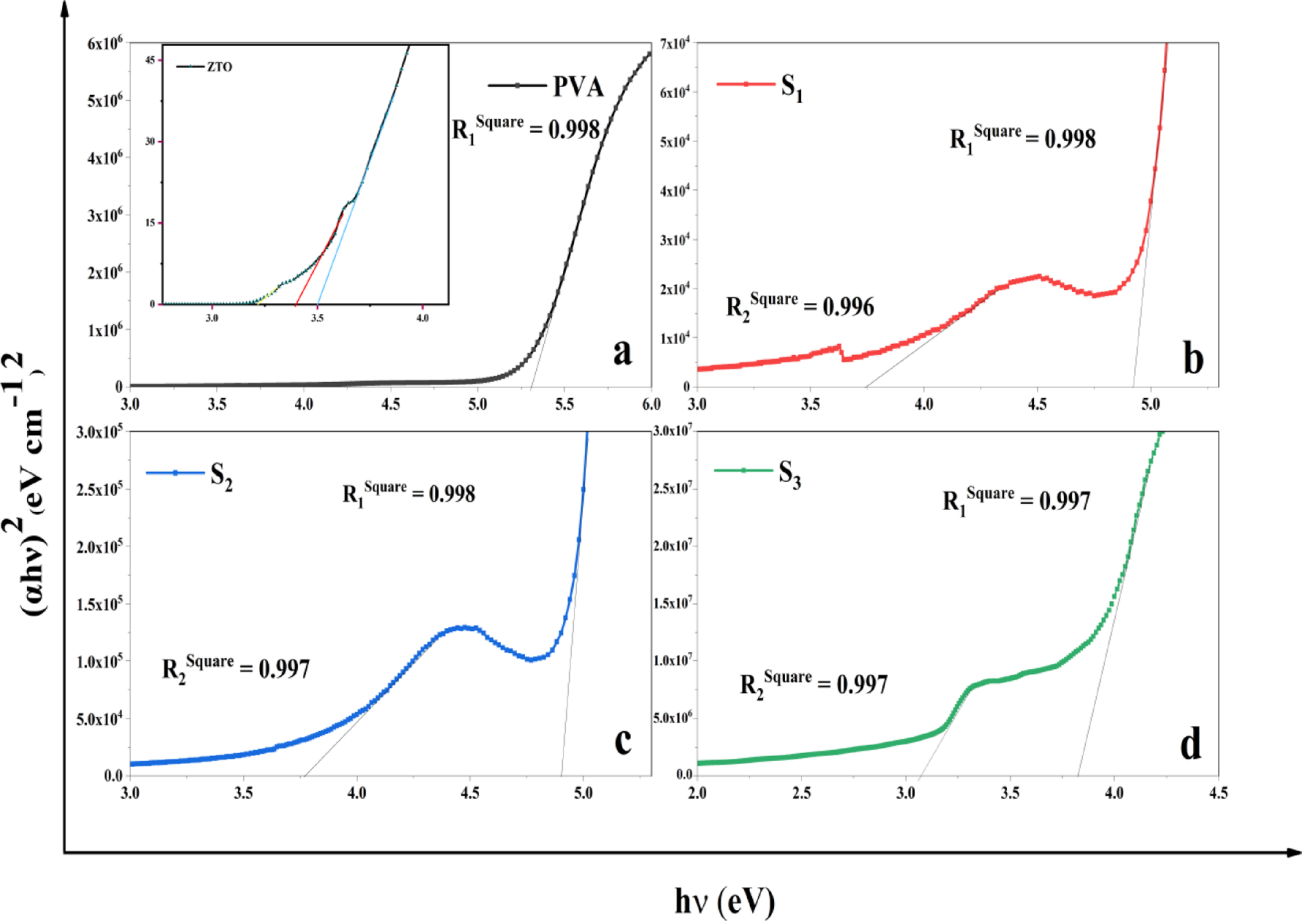
Tauc plots for direct allowed transitions: (**a**) Pure PVA (inset: ZTO-NPs), (**b**) S_1_ (5% ZTO), (**c**) S_2_ (8% ZTO), and (**d**) S_3_ (10% ZTO), showing progressive reduction in optical bandgap.

Tauc plots for each sample are illustrated in Fig. 6, with linear extrapolation of the absorption edge determining E_**g**_ values. Two key spectral regions were identified: 210–240 nm (region 1) and 290–340 nm (region 2), representing absorption transitions from the PVA matrix and ZTO nanoparticles, respectively. The quality of linear fits assessed was using coefficient of determination R-square (R^**2**^) values^52,53^.

#### Primary bandgap (PVA matrix)

The primary E_**g**_, attributed to the PVA matrix, exhibits a clear decreasing trend with increased ZTO loading (Pure PVA: 5.28 eV; S1: 4.92 eV; S2: 4.89 eV; S3: 3.81 eV). This reduction can be ascribed to some reasons including; Interfacial Defect States: Incorporation of ZTO-NPs introduces localized states within the PVA band structure, facilitating lower-energy electronic transitions^54^; Enhanced Charge Transfer: Interaction between polymer chains and ZTO facilitates delocalization of charge carriers, effectively narrowing E_g_^55^; and Structural Disorder: The inclusion of ZTO-NPs disrupts local polymer ordering and introduces tail states, shifting the absorption edge to longer wavelengths (red shift)^56,57^. These combined effects result in a systematic modulation of the electronic structure of PVA through nanoparticle-induced hybridization.

#### Secondary bandgap (ZTO contribution)

The secondary E_**g**_, arising from the ZTO nanofiller, displays a non-monotonic trend with increasing nanoparticle concentration (S1: 3.73 eV; S2: 3.76 eV; S3: 3.06 eV). This behavior can be interpreted via three key mechanisms: first quantum confinement at low concentrations: In S_**1**_ and S_**2**_, well-dispersed nanoparticles retain quantum size effects, marginally increasing the bandgap due to confined electronic states^58^. Second agglomeration effects at high concentration: In S_3_, substantial nanoparticle agglomeration increases the effective particle size, diminishing confinement and resulting in E_g_ narrowing^59^.Third defect state formation: Higher ZTO content introduces additional oxygen vacancies and surface defects, generating mid-gap states that reduce the apparent optical bandgap^60^.

Figure 6a inset highlights these variations through direct allowed Tauc plots. Notably, three absorption bands at 3.21 eV, 3.39 eV, and 3.49 eV are observed, corresponding to ZnO, SnO_**2**_, and Zn_**2**_SnO_**4**_ phases, respectively confirming the composite nature of the optical response.

A summary of the extracted E_**g**_ values is provided in Table 2:

**Table 2:** Optical bandgap (E_**g**_) values for direct allowed transitions in pure PVA and ZTO/PVA nanocomposite samples.

Sample	Bandgap 1 (eV)	Bandgap 2 (eV)
PVA	5.28	–
ZTO-NPs	–	3.49
S_1_	4.92	3.73
S_2_	4.89	3.76
S_3_	3.81	3.06

The observed dual bandgap behavior underscores the synergistic interaction between the polymeric host and the ZTO filler. While the primary bandgap (PVA-related) steadily decreases with higher ZTO-NPs content, the secondary E_**g**_ (ZTO-related) varies due to competing effects of quantum confinement and particle agglomeration.

These findings illustrate the potential to tune the optical bandgap of PVA through ZTO loading, providing an effective strategy for engineering nanocomposites for UV filtering, optical sensing, and flexible optoelectronic devices^61,62^.

### QCM-based detection of arsenic using ZTO-NPs

Gold-coated AT-cut quartz crystals (5 MHz fundamental frequency, Biolin Scientific) were used as QCM electrodes. The electrodes were first cleaned with freshly prepared piranha solution (3:1 H_**2**_SO_**4**_:H_**2**_O_**2**_) to remove organic contaminants, following established protocols^63^. After cleaning, the electrodes were rinsed thoroughly with ultrapure deionized water (18.2 MΩ cm) and dried under a stream of high-purity nitrogen gas.

For each nanocomposite formulation S_**1**_ (5 wt% ZTO/PVA), S_**2**_ (8 wt% ZTO/PVA), and S_**3**_ (10 wt% ZTO/PVA) a 1 mg·mL^−1^ suspension in ethanol was prepared by dispersing the dried nanocomposite powder using probe sonication (20 kHz, 60% amplitude, 10 min, pulse mode) to ensure homogeneity. Twenty microliters of the respective suspension was drop-cast onto the cleaned electrode surface, ensuring complete coverage of the active gold area, and allowed to dry under ambient conditions (25 °C, 40–50% RH) for 24 h to promote uniform film formation.

The final dried coating thicknesses were measured using a stylus profilometer, and averaged 1.20 ± 0.10 µm (S_**1**_), 1.40 ± 0.10 µm (S_**2**_), and 1.60 ± 0.10 µm (S_**3**_). Thickness consistency was confirmed by measuring three random points on each electrode, with variations below 5%. Coating uniformity was verified by optical microscopy, confirming continuous coverage without visible pinholes or cracks.

In parallel, the areal mass loading (Γ) was determined from the Sauerbrey equation using the measured frequency shift of the fundamental resonance after coating^64^. The calculated loadings were 268 µg·cm^−2^ (S_**1**_), 323 µg·cm^−2^ (S_**2**_), and 377 µg·cm^−2^ (S_**3**_), corresponding to equivalent compact thicknesses of 1.20 ± 0.10 µm, 1.40 ± 0.10 µm, and 1.60 ± 0.10 µm, respectively, assuming composite densities obtained from bulk cast-film measurements. These values were in excellent agreement with the profilometer results. All coatings exhibited low dissipation (ΔD < 1 × 10^−6^), confirming operation in the Sauerbrey regime.

Arsenic sensing experiments were performed using a QCM-D system (Q Sense, Biolin Scientific) under controlled environmental conditions (25.0 ± 0.1 °C and 60 ± 5% RH). Measurements were conducted at a flow rate of 100 µL·min^−1^, and tests were performed across different pH levels (3, 7, and 9) and arsenic concentrations (1, 5, and 10 ppm). Frequency (**Δ**f) and dissipation (**Δ**D) shifts were recorded at 5 min intervals over a 30 min exposure period. Each experiment was conducted in triplicate to ensure statistical reliability, following established QCM protocols^65^.

#### pH-dependent sensing characteristics

**Fig. 7:**
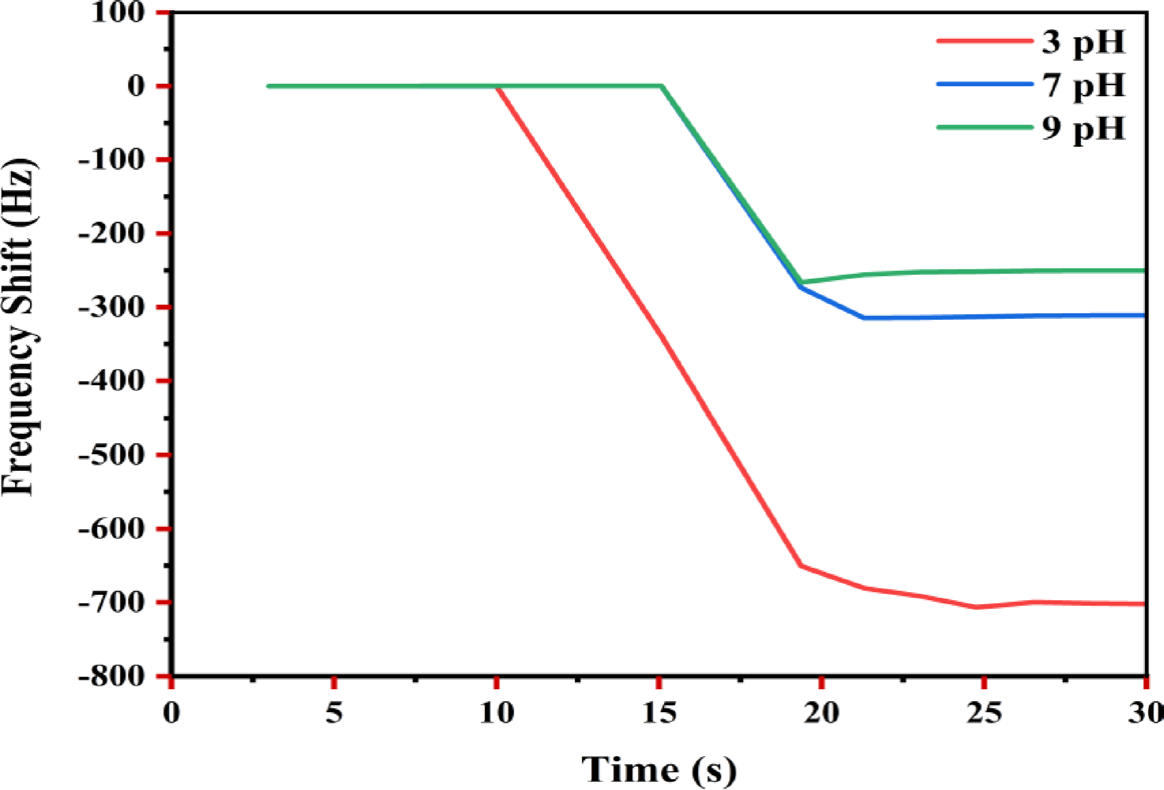
Real time QCM response for As (III) detection at different pH values.

The pH-dependent sensing performance of S_**1**_, S_**2**_, and S_**3**_ was evaluated toward As (III) detection at pH 3, 7, and 9. Real-time frequency monitoring revealed distinct pH-dependent behavior, consistent with previous observations^66^. Figure 7 illustrates the real-time QCM sensor responses for As (III) detection under varying pH conditions**.**

- Initial response time: The fastest responses occurred at pH 3 (~ 10 s), followed by pH 7 and pH 9 (~ 15 s). The enhanced responsiveness under acidic conditions is attributed to stronger electrostatic and surface complexation interactions between As (III) species and the ZTO surface^66,67^.
- Frequency shift magnitude: Maximum frequency shifts followed the order pH 3 (~ 700 Hz), followed by pH 7 (~ 310 Hz) and pH 9 (~ 250 Hz). This trend, illustrated in Fig. 7 and summarized in Table 3, consistent with arsenic speciation and adsorption efficiency under different pH conditions^68^.
- Stability and reproducibility: The relative standard deviation (RSD) values were 1.2% (pH 3), 0.8% (pH 7), and 1.5% (pH 9), indicating good reproducibility across all conditions, with pH 7 demonstrating the highest stability. These results are consistent with other metal oxide based sensing systems^69^.
- Sensitivity: Sensitivity, defined as the frequency shift per ppm arsenic, was highest at pH 3 (70.64 Hz/ppm), followed by pH 7 (32.38 Hz/ppm) and pH 9 (26.42 Hz/ppm). This trend is attributed to surface charge interactions and arsenic speciation under varying pH conditions, as also discussed in^69^.
- Long-term stability: While frequency responses plateaued in all cases, the sensor at pH 3 exhibited slight drift over time, likely due to mild acidic corrosion, consistent with earlier reports^70^.

**Table 3:** ph-dependent sensing characteristics of ZTO-NPs coated QCM sensors.

pH	Initial response time (s)	Max frequency shift (Hz)	Stability (RSD %)	Sensitivity (Hz/ppm)
3.0	~ 10	~ 700	1.2	70.64
7.0	~ 15	~ 310	0.8	32.38
9.0	~ 15	~ 250	1.5	26.42

These results indicate that pH 3 provides optimal sensitivity and response speed for As(III) detection using ZTO/PVA nanocomposites, while pH 7 offers the best balance between stability and sensitivity^71,72^.

#### Concentration-dependent sensing characteristics

**Fig. 8:**
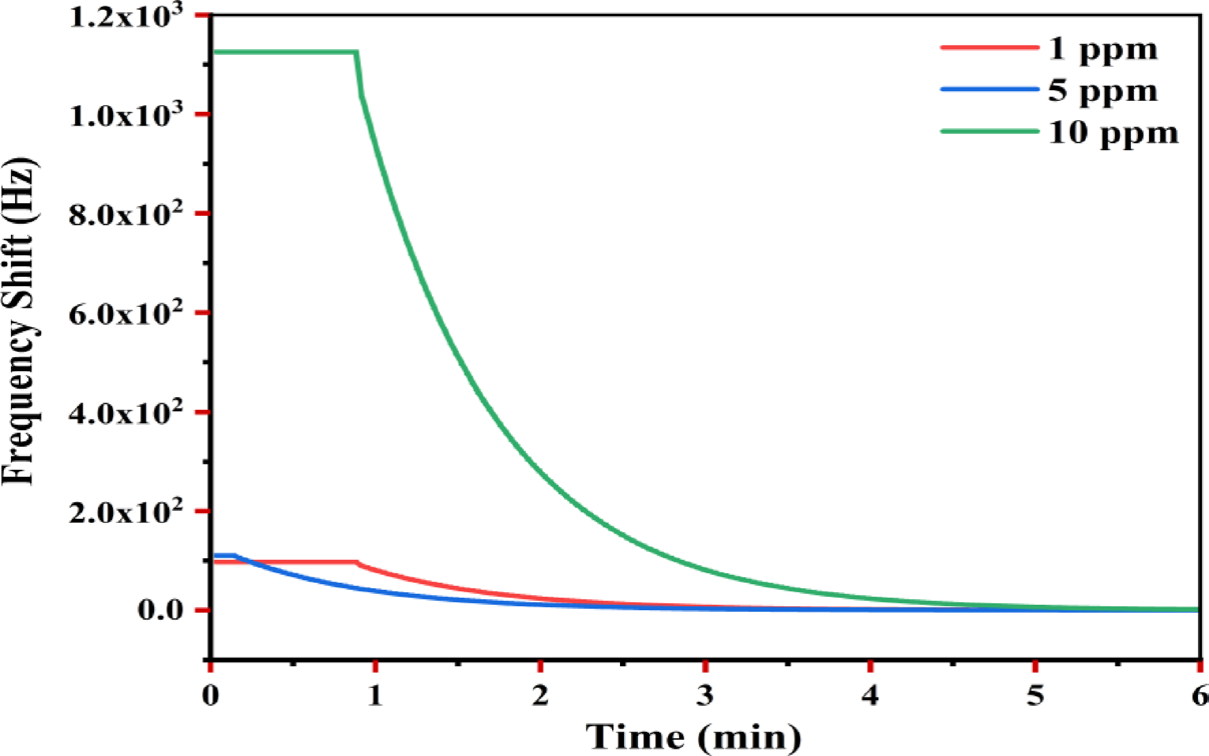
Real time QCM response at different As (III) concentrations.

Concentration dependent performance was investigated for As (III) at pH 3 (optimal pH) with concentrations of 1, 5, and 10 ppm (Fig. 8, Table 4), following the procedures outlined in^73^.

- Initial response time: The sensor response times decreased with increasing concentration: ~ 0.9 min at 1 ppm, ~ 0.2 min at 5 ppm, then increasing slightly to ~ 0.8 min at 10 ppm. This trend indicates faster sensor activation at intermediate analyte levels, consistent with kinetic adsorption models where initial high-concentration exposure accelerates surface occupation^74^.
- Frequency shift: As illustrated in Table 4**,** the frequency shift increased nonlinearly with concentration: ~ 110 Hz at (1 ppm), ~ 120 Hz at (5 ppm), and then jump to ~ 1130 Hz at (10 ppm). The large jump at 10 ppm suggests cooperative adsorption or partial surface saturation effects^75^.
- Response kinetics: Steady-state was reached at ~ 3.5 min for both 1 ppm and 5 ppm, while 10 ppm required ~ 4.0 min. These minor variations support the view that adsorption kinetics remain rapid even at high surface coverage, but may be influenced by aggregation or reorganization processes at elevated analyze loadings^76^.
- Sensitivity: Sensitivity values also exhibited a non-linear trend: 110 Hz ppm^−1^ at 1 ppm, decreasing to 24 Hz ppm^−1^ at 5 ppm, and increasing again to 113 Hz ppm^−1^ at 10 ppm. At low As (III) concentrations, the sensor surface offers abundant high-energy adsorption sites (e.g., hydroxylated ZTO terminations, defect sites) where binding is strong and rapid, yielding a high frequency shift per unit concentration. At intermediate concentrations (5 ppm), these sites become progressively saturated, forcing adsorption to occur on lower-energy sites with weaker binding affinity, leading to a smaller incremental frequency shift and lower apparent sensitivity. At higher concentrations (10 ppm): the adsorption regime likely transitions from predominantly monolayer coverage to a mixed regime involving multilayer adsorption, surface precipitation of arsenic species, or cooperative effects such as electrostatic clustering. Under these conditions, localized aggregation of As (III) and formation of surface complexes contribute disproportionately to the sensed mass per unit concentration, causing the marked sensitivity increase^77^. Such non-linear sensitivity profiles have been reported for other metal-oxide-based aqueous sensors, where the interplay between surface chemistry, analyze speciation, and adsorption kinetics dictates the response. The presence of pH-dependent arsenic species and surface hydroxyl groups on ZTO may further modulate these interactions, amplifying deviations from a simple linear concentration–response relationship^78^.
- Stability: Despite rapid initial shifts, the final stable response was lower at 5 ppm (~ 5 Hz) than at 1 ppm (~ 10 Hz) and 10 ppm (~ 30 Hz), further supporting the nonlinear interaction dynamics.

**Table 4:** Concentration dependent sensing characteristics at pH 3.

As(III) concentration (ppm)	Initial response time (min)	Max frequency shift (Hz)	Steady-state time (min)	Sensitivity (Hz/ppm)	Final stable response (Hz)
1	~ 0.9	~ 110	~ 3.5	110	~ 10
5	~ 0.2	~ 120	~ 3.0	24	~ 5
10	~ 0.8	~ 1130	~ 4.0	113	~ 30

ZTO modified QCM sensor demonstrated robust sensitivity across a broad concentration range, with particularly enhanced response at higher arsenic levels. The nonlinear response profile suggests complex adsorption mechanisms, including multilayer adsorption or sensor surface modifications, as supported by prior theoretical studies.

The ZTO-NPs modified QCM sensor exhibits both pH and concentration dependent sensing characteristics for As (III), with optimal performance observed at pH 3 and elevated arsenic levels. The sensor combines high sensitivity, rapid response, and acceptable reproducibility, supporting its potential for practical environmental monitoring of arsenic contamination. For applications prioritizing operational durability, neutral pH (7.0) presents a favorable trade-off between sensitivity and long-term stability, in agreement with recent comparative studies^79,80^.

#### Selectivity and sensitivity analysis

**Fig. 9:**
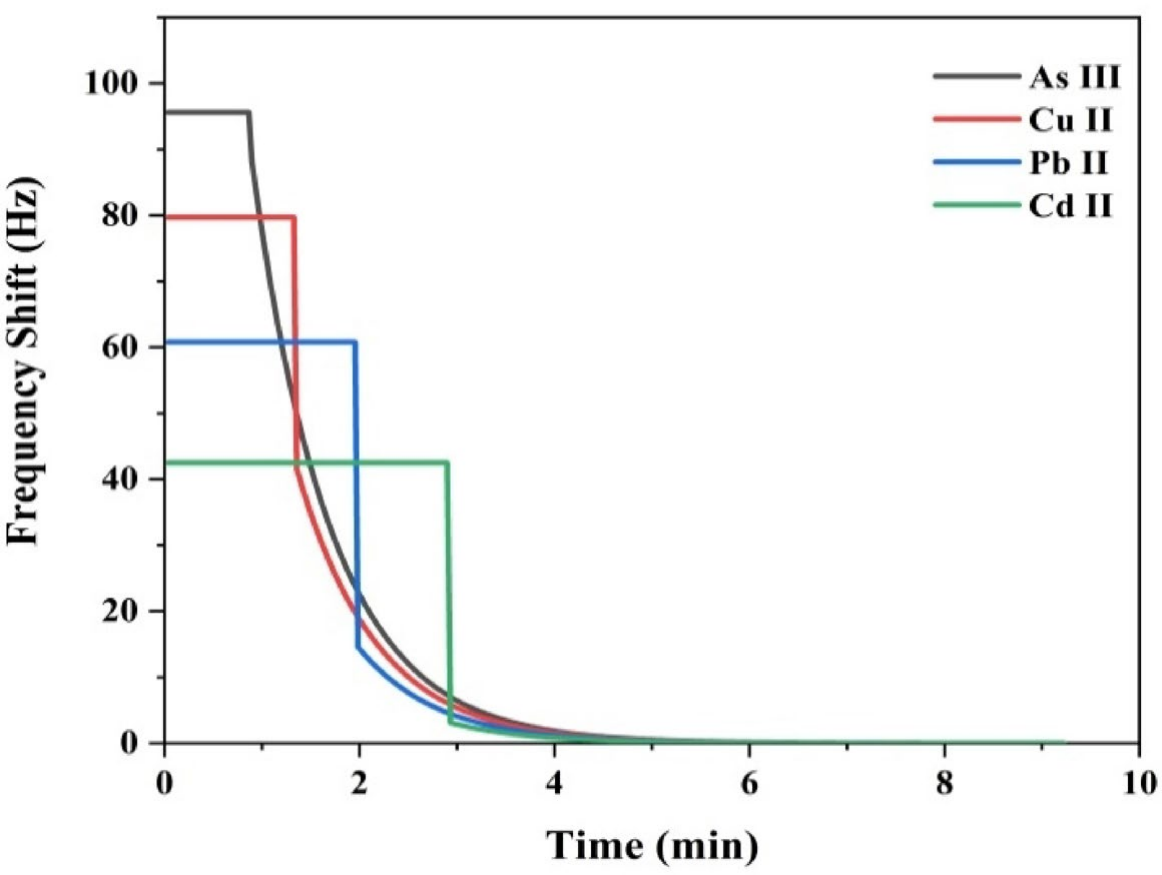
Selective frequency shift responses of the ZTO-NPs–modified QCM sensor toward As (III), Cu (II), Pb (II), and Cd (II).

The selectivity of the ZTO-NPs modified QCM sensor was evaluated against four toxic metal ions As (III), Cu (II), Pb (II), and Cd (II) each tested at comparable concentrations. The frequency shift curves in Fig. 9 demonstrate the pronounced preference of the sensor for As (III) detection over the other tested heavy metal ions.

The ZTO-NPs sensor exhibited the highest initial frequency shift toward As (III), reaching approximately 95 Hz within the first minute of exposure. This response is ~ 19% higher than Cu (II), ~ 53% higher than Pb (II), and ~ 121% higher than Cd (II). The rapid and substantial frequency shift reflects a strong binding affinity between ZTO-NPs and As (III) species, attributable to surface complexation and the presence of high-affinity sites on the Zn-Sn oxide structure^81,82^. Nanostructured sensing materials often provide superior selectivity due to their high surface area, abundant active sites, and tunable surface chemistry^83^.

The sensor’s frequency shifts for the other ions were significantly lower Cu (II): 80 Hz, Pb (II): 60 Hz and Cd (II): 42 Hz demonstrating a clear selectivity order:

$${\text{As}}\left( {{\text{III}}} \right)\, > \,{\text{Cu }}\left( {{\text{II}}} \right)\, > \,{\text{Pb }}\left( {{\text{II}}} \right)\, > \,{\text{Cd }}\left( {{\text{II}}} \right)$$

While Cu (II) exhibited moderate interaction with the ZTO surface, Pb (II) and Cd (II) showed progressively weaker affinities. Given the environmental toxicity of these ions^84^, the ability to discriminate between them is essential for targeted contamination monitoring Table 5.

**Table 5:** Shows relative selectivity, responses were normalized to the maximum observed signal (As (III) = 1.00).

Ion	Initial frequency shift (Hz)	Normalized response (Relative to As(III))	Stabilization time (min)
As (III)	~ 95	1.00	~ 1
Cu (II)	~ 80	0.84	~ 1.5
Pb (II)	~ 60	0.65	~ 2
Cd (II)	~ 42	0.45	~ 3

a *Temporal response characteristics* The ZTO/PVA-QCM sensor exhibited strong selectivity for As (III), producing the highest signal magnitude and achieving rapid stabilization (~ 1.5 min) compared to Cu (II), Pb (II), and Cd(II) (which required ~ 2–3 min). This fast and robust response is attributed to As (III)’s favorable ionic radius (~ 0.58 Å), compatible coordination geometry, and oxidation state, which enable strong electrostatic and covalent interactions with the oxygen-rich, amphoteric surface of Zn–Sn oxide^85^. These properties promote selective chemisorption and the formation of stable complexes, resulting in a 2.4-fold higher signal for As (III) over Cd (II), even in multi-ion environments highlighting the sensor are potential for real-world arsenic monitoring in contaminated water^81^.

While demonstrating excellent short-term stability and reproducibility, especially at neutral pH, prolonged exposure to acidic conditions may gradually degrade the sensing layer. Future work will focus on evaluating operational lifespan, cycling stability, and regeneration protocols to ensure long-term reliability under various pH conditions, particularly acidic environments where sensitivity is maximized. Comprehensive interference studies involving common anions (phosphate, sulfate, nitrate, chloride) and cations present in drinking water, along with tests in real environmental samples, are also planned to validate specificity and practical applicability in complex water matrices.

#### Effect of ZTO wt% on sensing performance

The sensing performance of the three ZTO/PVA-modified QCM sensors (S_**1**_, S_**2**_, and S_**3**_) revealed clear trends related to the ZTO nanoparticle loading. Increasing the ZTO wt% generally improved areal mass loading, coating thickness, and surface coverage, which in turn enhanced the active surface area available for As (III) adsorption.

Under optimal sensing conditions (pH 3), S₃ exhibited the highest maximum frequency shift and sensitivity, followed by S_**2**_, with S_**1**_ showing the lowest values. This improvement is attributed to the increased density of ZTO active sites in higher wt% coatings. However, the higher thickness in S_**3**_ (1.6 µm) also resulted in slightly slower response stabilization compared to S_**1**_ and S_**2**_, likely due to diffusion limitations in thicker films.

In terms of reproducibility, all sensors showed low RSD values (< 1.5%), but S₂ demonstrated the best stability sensitivity balance, suggesting that an intermediate ZTO wt% may be optimal for applications prioritizing long-term measurement reliability. These findings indicate a trade-off between sensitivity and dynamic response, controlled by the ZTO nanoparticle content (Table 6**)**.

**Table 6:** Performance comparison of ZTO/PVA sensors at pH 3.

Parameter	S₁ (5 wt% ZTO/PVA)	S₂ (8 wt% ZTO/PVA)	S₃ (10 wt% ZTO/PVA)	Trend with ↑ ZTO wt%
Coating thickness (µm)	1.20 ± 0.10	1.40 ± 0.10	1.60 ± 0.10	↑ with wt%
Areal mass loading (µg·cm^−2^)	268	323	377	↑ with wt%
Max frequency shift (Hz)	~ 520	~ 640	~ 700	↑ with wt%
Sensitivity (Hz/ppm)	52.3	64.2	70.6	↑ with wt%
Initial response time (s)	~ 9	~ 10	~ 10	Slight ↑ for higher wt%
Stability (RSD %)	1.0	0.8	1.2	Min at mid wt%
Steady-state time (min)	~ 3.0	~ 3.5	~ 4.0	↑ for higher wt%

Limit of detection (LOD) and Limit of quantification (LOQ)

LOD and LOQ were determined according to the IUPAC definitions:

9 $${\text{LOD}} = \frac{{3.3 \times \sigma_{blank} }}{S}$$

10 $${\text{LOQ}} = \frac{{10\user2{ } \times \user2{ }\sigma_{blank} }}{S}$$

where S is the sensor sensitivity (Hz·ppm^−1^) and $\sigma_{blank}$ is the standard deviation of the blank signal. Since dedicated blank-only measurements were not conducted in this work, $\sigma_{blank}$ was conservatively estimated from the reproducibility (relative standard deviation, RSD) of the maximum frequency shift obtained in triplicate:

11 $$\sigma_{blank} \approx \frac{RSD \% }{{100}}\; \times \;\Delta \;f_{\max } \user2{ }$$

Using values reported in Table 6. The calculated LOD and LOQ values for ZTO-NPs at pH 3 are summarized in Table 7, showing LODs between 0.24:0.36 ppm and LOQs between 0.80:1.19 ppm. These results confirm that the proposed sensors can quantify arsenic at the sub-ppm level; however, the current detection limits remain above the WHO guideline value for drinking water (10 ppb).

**Table 7:** Calculated LOD and LOQ values for ZTO-NPs at pH 3.

Sample	Sensitivity S (Hz·ppm^−1^)	${\Delta f}_{{{\text{max}}}}$(Hz)	RSD (%)	σ_blank_ (Hz)	LOD (ppm)	LOQ (ppm)
S₁ (5 wt%)	52.3	520	1.0	5.20	0.33	0.99
S₂ (8 wt%)	64.2	640	0.8	5.12	0.26	0.80
S₃ (10 wt%)	70.6	700	1.2	8.40	0.39	1.19

The variation of LOD and limit of LOQ with increasing ZTO-NPs concentration in samples S_**1**_, S_**2**_, and S_**3**_ shows a characteristic (${\text{decrease}}\; \to Then \to \;{\text{increase}}$) behavior. At lower ZTO-NPs loadings, the increasing of nanoparticle concentration improves the sensitivity of the sensor due to the higher availability of active surface sites for adsorption of the target analyze. This enhanced sensitivity leads to a reduction in both LOD and LOQ, as smaller analyze concentrations can be detected and quantified reliably.

However, beyond an optimal nanoparticle loading, further increase in ZTO-NPs concentration results in aggregation of nanoparticles and possible partial blocking of the sensor’s active surface. This can reduce the effective surface area, hinder analyze diffusion, and increase baseline noise, thereby reducing sensitivity. As a result, both LOD and LOQ begin to increase again at higher loadings^86^.

This behavior indicates that there is an optimal ZTO-NPs concentration at which the sensor achieves its best analytical performance, characterized by the lowest LOD and LOQ. Below or above this concentration, performance deteriorates due to insufficient active sites (low loading) or surface saturation/aggregation effects (high loading), consistent with trends reported in other nanoparticle-modified sensing systems^81,82,86^.

## Conclusion

This work presents a comprehensive study on the development and performance of ZTO/PVA nanocomposites for arsenic detection, revealing significant correlations between material compositions, structure, and sensing behavior. The successful hydrothermal synthesis of phase-pure cubic ZTO-NPs and their incorporation into PVA matrices at varying concentrations S_**1**_, S_**2**_, and S_**3**_ led to nanocomposites with tunable physical and chemical properties.

Structural analysis via XRD confirmed the formation of crystalline ZTO with a preferred (311) orientation and revealed concentration-dependent changes in crystallite size, lattice strain, and unit cell parameters. Raman spectroscopy further validated the integrity of the inverse spinel phase and indicated polymer nanoparticle interactions. SEM analysis demonstrated increased surface roughness and nanoparticle aggregation at higher ZTO loadings, which affected the morphological uniformity of the films.

Optical studies revealed dual-bandgap behavior, with the primary bandgap systematically decreasing from 5.28 eV (pure PVA) to 3.81 eV (S_**3**_), suggesting enhanced charge transfer interactions between the polymer and filler. The tunability of these optical properties extends the potential application of these composites beyond sensing to optoelectronics and UV shielding.

The QCM based sensing studies demonstrated excellent arsenic detection performance, particularly under acidic conditions (pH 3), where the highest sensitivity (70.64 Hz/ppm) and fastest response time (~ 10 s) was recorded. The non-linear concentration dependent response, with peak sensitivity of 113 Hz/ppm at 10 ppm, suggests complex adsorption dynamics, potentially involving multilayer or cooperative binding mechanisms.

These findings position ZTO/PVA nanocomposites as promising candidate for high-performance environmental sensors targeting arsenic contamination. The current ZTO/PVA-QCM platform demonstrates rapid, selective, and reproducible detection, making it well-suited for arsenic screening in scenarios with elevated contamination, despite its present detection limit (~ 1 ppm) being above the WHO guideline of 10 ppb for drinking water. The strong As (III) selectivity, short response time, and stability highlight its potential for practical deployment in environmental monitoring.

Future research will focus on advancing the platform toward trace-level detection through surface functionalization, nanostructure optimization, and pre-concentration strategies, as well as mechanistic modeling of sensor analyze interactions. Comprehensive real-world testing in diverse water matrices, alongside further interference studies will ensure specificity and reliability under complex conditions. By addressing these aspects, the ZTO/PVA-QCM sensor can evolve into a next-generation sensing platform for public health protection and environmental safety.

## Data Availability

The datasets generated and/or analyzed during the current study are available from the corresponding author, and author on reasonable request. Requests for data should be directed to Prof. Dr. Mohamed Mounir, and Dr. Ahmed Samir.
